# Physical Activity and Nutrition Intervention for Middle Schoolers (Move More, Get More): Protocol for a Quasi-Experimental Study

**DOI:** 10.2196/37126

**Published:** 2022-05-04

**Authors:** Amanda Grimes, Joseph S Lightner, Katlyn Eighmy, Bridget D Wray, Ella Valleroy, Maya Baughn

**Affiliations:** 1 School of Nursing and Health Studies University of Missouri Kansas City, MO United States; 2 Department of Population Health University of Kansas Medical Center Kansas City, KS United States; 3 School of Urban Planning University of Kansas Lawrence, KS United States

**Keywords:** intervention protocol, physical activity, food intake, nutrition, healthy eating, middle schoolers, youth, school, student, fitness, exercise, food consumption, diet, fruit consumption, vegetable consumption

## Abstract

**Background:**

Physical activity and nutrition behaviors are important to reducing the prevalence of childhood obesity. Previous research has identified school-based interventions as effective strategies to improve physical activity and nutrition. However, the results are often mixed, and middle schoolers are an under-studied population.

**Objective:**

Our study aims to fill this gap by developing an after-school intervention to increase physical activity and fruit and vegetable consumption that is influenced by national guidelines and formative research.

**Methods:**

This study was an after-school, quasi-experimental study spanning 9 months. Enrollment began in September 2021 and continued on a rolling basis through February 2022. Weekly, middle schoolers were offered 2-3 physical activity sessions and 1 produce kit. Physical activity was measured using accelerometers and questionnaires. Nutrition behaviors were assessed using questionnaires, and physical literacy was assessed using researcher observations. Follow-up data collection occurred in December 2021 and in April 2022. Difference scores will be calculated and analyzed for each outcome variable.

**Results:**

The intervention started in September 2021 and will conclude in May 2022. Published study results are expected in late 2022.

**Conclusions:**

An increase in physical literacy, physical activity, and fruit and vegetable consumption is expected. If successful, future studies will focus on reach and sustainability. Lastly, this study may serve as a model for improving health outcomes in middle schools.

**International Registered Report Identifier (IRRID):**

DERR1-10.2196/37126

## Introduction

Physical inactivity and poor nutrition are strong predictors of negative health status and increase the risk for obesity and other chronic diseases [1]. In the United States, the majority of youth do not meet the national guidelines for physical activity [2] or nutrition [3]. Among middle schoolers—children in 6th to 8th grades—71.3% did not meet the physical activity recommendation of 60 minutes per day of moderate-to-vigorous physical activity [2]. Evidence suggests that the COVID-19 pandemic had additional negative impacts on middle schoolers’ physical activity [4]. National guidelines recommend that children and adolescents consume 2.5 cups of vegetables and 2 cups of fruit per day [5]. However, only 7.1% and 2% of adolescents met the United States Department of Agriculture recommendations for fruit and vegetable consumption, respectively [3]. Consequently, obesity has risen to 21.2% among children and adolescents aged 12-19 years in 2018 compared to 18.4% in 2010 [6].

Schools are an ideal place to promote physical literacy [7] and physical activity to help students achieve physical activity recommendations [8-11], and physical activity in after-school programming is an effective strategy to increase youth physical activity [12-14]. Programming should focus on developing the foundational skills necessary to participate in a variety of activities, as youth who are more physically literate are more likely to be active throughout life [15]. Moreover, evidence suggests that middle schoolers prefer programming that incorporates a variety of sports [16]. Interventions that incorporate sports sampling—the practice of participating in a variety of sports—promote physical activity through adolescence and into adulthood [17,18].

A systematic review by Patrick and Nicklas [19] found that family environment and the availability of fruits and vegetables are strong predictors of fruit and vegetable consumption. However, evidence suggests that school-based nutrition interventions have mixed or limited success in improving fruit and vegetable consumption [20-22] or decreasing BMI [22]. For example, Davis et al [22] found that a gardening, cooking, and nutrition intervention significantly increased vegetable intake, but there was no impact on fruit consumption or BMI. A review conducted by Dabravaolskaj et al [20] found that modifications to school nutrition policies resulted in significant positive consumption for fruit, but not for vegetables. The findings from a systematic review of school-based nutrition interventions suggest that no dominant factor was shared among studies with significant findings [21].

There is convincing evidence that suggests school-based interventions to reduce obesity are effective; however, evaluations of the factors contributing to effectiveness are inconclusive [23,24]. Therefore, the purpose of this study is to describe the study protocol used to evaluate the effectiveness of an after-school intervention on increasing physical activity and fruit and vegetable consumption among middle schoolers in an urban Midwestern US school district.

There are two primary hypotheses for this study. (1) The intervention group will show consecutive increases in physical literacy and physical activity from the baseline in 2 follow-up tests compared to the control group. (2) The intervention group will show consecutive increases in fruit and vegetable consumption from the baseline in 2 follow-up tests compared to the control group. Our secondary hypothesis posits that positive changes in physical literacy and physical activity will be mediated by the intervention dose.

## Methods

### Study Design

A two-arm, quasi-experimental study was conducted. In an urban Midwestern US public school district, 3 middle schools were identified by the district administration to be included as intervention schools in this study based on need and student population. An additional middle school in the same city with a demographically similar student population was selected to participate as a control. All middle schoolers at the intervention schools were invited to participate in the study. Data were collected during the after-school programming at 3 time points during the academic school year: September 2021, December 2021, and April 2022. All middle schoolers at the control school were invited to participate in a physical activity and nutrition research study. After parental consent and upon enrollment, informed assent was obtained from the middle schooler–participants. The participants then completed a questionnaire assessing demographics, physical activity, and nutrition. Additionally, the participants’ weight and height were assessed by research staff to calculate their BMI. Research staff also assessed the participants’ physical literacy using the validated PLAYBasic instrument [25,26]. Lastly, all participants were instructed to wear a Garmin Vivofit 4 (Garmin International) accelerometer to track steps and the duration of physical activity for the remainder of the study period. Although moderate- to vigorous-intensity reliability and validity measures were not available for the Garmin Vivofit 4, Garmin wearables are found to be valid and reliable for measuring steps [27]. Coupled with the high acceptability [28] and affordability of the Garmin Vivofit 4, we found it to be a good fit for this study.

Middle schoolers in the intervention group were offered a sport sampling program after school each week throughout the school year. The number of sessions offered was determined by the school’s after-school programming schedule. The sport-sampling programming was facilitated by trained coaching staff and rotated sports every 2 weeks. Sport training focused on developing foundational skills and physical literacy and concluded with scrimmaging. Middle schoolers in the intervention group were also offered a weekly distribution of produce. To reduce barriers to participation, the schools offered free transportation home for participants in after-school activities. Middle schoolers in the control group were asked to continue with their regular routines. All participants (intervention and control) were asked to download the Garmin Connect app, given a research team–developed login, and taught how to sync their accelerometer device. Research staff was available to help troubleshoot syncing issues and sync the participants’ accelerometers during school visits if they were unable to sync their accelerometer using a personal smartphone or tablet. All participants repeated the baseline testing after approximately 3, 6, and 9 months.

### Ethics Approval

All study procedures were approved by the University of Missouri-Kansas City Institutional Review Board (#2017528).

### Participant Recruitment

All middle schoolers at the participating schools were eligible to participate in the study. Recruitment began in September 2021 and continued on a rolling basis through February 2022. Recruitment efforts required a multimodal strategy: direct recruitment, snowball sampling, and referral recruitment. Research staff attended school lunches and district enrollment events to directly enroll students. Financial giveaways aided enrollment interest during in-person recruitment. Students had the opportunity to aid researchers by recommending peer groups for recruitment or referring friends directly to the program.

### Power Analysis

The sample size needed to understand if the intervention will have an effect was calculated using G*Power (version 3.1.9.4; Heinrich-Heine-Universität Düsseldorf) [29]. A systematic review of physical activity interventions suggested that similar interventions have significant but small effect sizes (0.44, 95% CI 0.19-0.70) [30]. A review of school-based nutrition interventions on behavior suggested that the average intervention has a small effect (0.33, 95% CI 0.55-1.10) on adolescent BMI [31].

Based on a *P* value of <.05 and physical activity interventions having a small effect size of 0.44 on outcome measures, this study requires a minimum of 47 participants to understand the differences between matched pairs for physical activity behavior. Based on a *P* value of <.05 and nutrition interventions having a small effect size of 0.33 on the consumption of fruits and vegetables, this study requires a minimum of 90 participants to understand the differences between matched pairs for fruit and vegetable consumption. We expected a 20% attrition that is similar to other school-based health behavior interventions [32]. Therefore, a minimum of 108 middle schoolers would need to be recruited through the participating schools (control and intervention).

### Description of the Intervention

#### Overview of the Program

“Move More, Get More” was an after-school intervention targeting physical activity and nutrition for urban middle schoolers at select middle schools in the Kansas City Public School District. The program was designed following national physical activity and nutritional guidelines and evidence from previous research. The programming was further influenced by the formative research conducted by the research team [16]. Key research findings centered on the promoters and barriers to physical activity and fruit and vegetable consumption, both before and during the COVID-19 pandemic. Parents and students from these focus groups made recommendations that guided program development. To increase physical activity, parents and students recommended opportunities for competition, goal setting, and financial incentives [16].

The program duration was set for 9 months, from September 2021 through May 2022, following Kansas City Public School District’s academic calendar. Baseline data were collected at the time of enrollment (from September 2021 to February 2022) and follow-up data collection occurred in December 2021 and April 2022. Session frequency and duration were dependent on each site’s after-school transportation availability and dismissal schedule. The session frequency and duration at the 3 sites were as follows: 3 sessions/week for 1-hour sessions, 3 sessions/week for 2-hour sessions, and 2 sessions/week for 1-hour sessions.

#### Physical Activity Programming

Sessions were designed to achieve 1 hour of moderate-to-vigorous physical activity daily, per the Centers for Disease Control and Prevention’s physical activity guidelines for youth [8] and were held at the students’ school. Sessions were hosted immediately after school in accordance with the school’s other after-school programs. Evidence from our formative research suggested that physical activity sessions would be more successful if they included opportunities for fun, peer influence, competition, goal setting, and incentives [16]. Additional findings suggested that time constraints, the overcompetitive nature of sports programs, and decreased motivation and access to physical activity were barriers to physical activity [16]. As a result, we designed the programming with the following aims: introduce a variety of sports and the skills necessary to participate in a variety of sports; encourage peer interaction by implementing snowball recruitment and focusing on team-oriented sports; provide opportunity for team competition through scrimmages and for goal setting and individual competition through step challenges using accelerometers. Incentives were used to increase motivation, encourage consistent participation, and facilitate thorough data collection. Furthermore, programming focused on skill development and inclusiveness and limited overcompetitiveness by implementing no-cut policies.

At the beginning of each semester, the program manager created a template schedule of sport and team activities, with activities rotating every 2 weeks. Activity types included traditional sports (basketball, soccer, football, etc), team-based activities (capture the flag, dodgeball, etc), dance, yoga, and others. This schedule was adapted by each intervention site’s coach based on their expertise, space, equipment availability, and student interest. For sport-based activities, fundamental and basic skills were taught during the sessions. During the last day of each unit, the participants would engage in scrimmage play. Each session included a 10-minute warm-up, an activity spanning 40-100 minutes, and a 10-minute cooldown.

Physical-activity coaches were primarily contracted through partnering organizations that are trusted and established within the school’s surrounding community (ie, community services center, parks and recreation department, and sport performance training center). Coaches were required to have previous experience leading youth physical activities. All staff completed mandated reporter training and a background check through the school district. Intervention sites were assigned 2 coaches and 2 or 3 researchers during each session. A staff-to–middle schooler ratio of 2 to 30 was required for all sessions.

#### Nutrition Programming 

Nutrition programming aimed to increase fruit and vegetable consumption toward meeting national guidelines. Furthermore, evidence from a systematic review suggested that family environment and availability are strong predictors of fruit and vegetable consumption [19] and cooking from home is associated with several nutritional benefits for youth [33]. Moreover, our previous findings suggested that parental control of nutrition behaviors and presentation, preparation technique, and convenience are all important factors to increasing fruit and vegetable consumption [16]. As a result, produce kits were distributed weekly to all middle schoolers.

Produce distributions were provided by University Health’s Healthy Harvest Mobile Market, a converted city bus designed to deliver fresh, healthy foods throughout Kansas City, Missouri. Each weekly distribution was procured to create at least one meal for a family of 5. Each bag included recipe staples (ie, onions, potatoes), recipe-specific produce, popular fruits, and occasionally unique fruits or vegetables middle schoolers may not ordinarily be exposed to. In addition to produce, University Health provided recipe cards, nutrition information, and food preparation techniques for at-home cooking. The produce bags were valued at approximately US $20.

#### Control Group 

Middle schoolers were invited to participate in the research study during physical education classes over a period of 2 weeks. Enrollment was conducted during each grade level’s physical education period over the span of 3 weeks. Control enrollment was identical to intervention data collection practices. All participants were asked to wear the accelerometer devices continuously throughout the study period. Participants in the control group were asked to continue their normal, routine activities. Control data collection took place during scheduled youth physical education classes.

### Incentives

Intervention participants received a US $25 gift card after completing baseline testing (questionnaire and objective measurements) and an additional US $25 gift card for each additional completed assessment at the 3- and 6-month time points. Participants in the control group received a US $10 gift card after completing baseline testing (questionnaire and objective measurements) and an additional US $10 gift card for each additional completed assessment at the 3- and 6-month time points. Since a participation component and larger time commitment was expected among the intervention group, we provided larger monetary incentives for the intervention group.

### Measures

#### Demographic Variables 

Middle schoolers’ demographic variables were assessed using a self-report questionnaire in the baseline test. Middle schoolers were asked their sex assigned at birth, birth date, race by selecting all that apply (American Indian or Alaska Native, Asian, Black or African American, Native Hawaiian or Other Pacific Islander, and White), and ethnicity (Hispanic or Latino) by indicating yes or no.

#### Physical Activity

Self-reported physical activity was assessed using the International Physical Activity Questionnaire Short Form, which assesses physical activity using a 7-day recall to estimate moderate- and vigorous-intensity physical activity, walking, and sedentary behavior [34]. All values were reported in minutes per week.

All middle schoolers were given a Garmin Vivofit 4 accelerometer to objectively measure physical activity throughout the study period. A valid day is defined as a middle schooler having ≥8 hours of wear time between 9 AM and 9 PM and ≥500 steps. To account for nonwear time, we considered 3 consecutive epochs of ≥15 minutes with a maximum motion intensity value of 0 as nonwear time. Daily data were aggregated at the weekly level for each participant; ≥1 valid day is required for the week to be included in the analyses. Garmin accelerometers automatically record moderate-to-vigorous physical activity; the feature is activated when the user walks for ≥10 minutes or runs for ≥1 minute.

Dietary Behaviors and Fruit and Vegetable Intake 

The selected questions were adapted from the 2019 National Youth Risk Behavior Survey for high schools that asked about fruit, vegetable, soda, and sport drink consumption in the past 7 days [35]. The original response options ranged on a 7-point scale from no consumption to >4 times a day; our survey collapsed the response options into a dichotomous yes-or-no format, as directed by the funding agency.

#### Height, Weight, and BMI

Height and weight were assessed objectively by trained research assistants using a validated scale [36] and stadiometer [37]. BMI was calculated with the following formula: *BMI = weight (kg)/ (height [m])^2^*.

#### Physical Literacy 

Trained research staff assessed physical literacy using the PLAYbasic instrument [26], which assesses the physical abilities of participants in 4 domains: balance, throwing, kicking, and locomotor. Staff asked the participants to perform 5 tasks: (1) run to a cone approximately 5 meters away, turn around, and run back to the starting point; (2) hop to the same cone on one leg, turn around, and hop back on the other leg to the starting point; (3) throw a tennis ball overhand to a wall 1.5 meters away and have it bounce back over their head; (4) kick a ball to a wall 4 meters away over a 1-meter line from the ground; and (5) walk backward toe to heel in a straight line for 2 meters. Each task was scored on a 0-100 scale with 0 being no proficiency and 100 being completely proficient. The scores were also categorized into the 4 rankings: initial (score of 0-25), emerging (score of 26-50), competent (score of 51-75), and proficient (score of 76-100); scores of 0-50 represent the developing rating, and scores of 51-100 represent the acquired rating. A final score was calculated by adding the section scores and then dividing by 5 according to the scale’s instructions [26].

### Statistical Analysis

SPSS Statistics for Windows (version 26; IBM Corp) will be used for data analysis. Univariate statistical analyses will be conducted for all study variables. Difference scores will be calculated for each outcome variable. Subsequently, a series of repeated measures analysis of covariance will be conducted to assess within-group differences, while controlling for school and other factors. To assess the dose response relationship between intervention attendance and the outcome variables, linear and logistic regression models will be conducted. An alpha level of 95% will be used for all analyses.

## Results

This study started in September 2021. Formative research to inform the intervention was conducted in December 2019 and from June to August 2020. The in-person intervention implementation was delayed until September 2021. As the study had a rolling enrollment period, we completed all baseline testing by November 2021. The results of the study will be communicated to the research and professional community via publications. We will communicate the results with other stakeholders (eg, community partners, parents, school staff, etc) via newsletters, social media posts, website, and local media.

## Discussion

### Expected Findings

As childhood obesity rates increase [5], it is important to expand access to noncompetitive, school-based physical activity programming and promote fruit and vegetable consumption. School-based interventions that reduce common barriers (eg, fees, transportation, competitiveness) are ideal to improving youth population–level health. Fostering foundational physical literacy skills [15] and increasing access to physical activity programming are necessary for youth to be and remain physically active through life [38] to prevent obesity and related chronic diseases [1]. In response, this study tested the effectiveness of the “Move More Get More” program for middle schoolers using a quasi-experimental study. More specifically, this study investigated whether the program could increase the middle schoolers’ physical literacy, physical activity, and fruit and vegetable consumption. Furthermore, we investigated the dose-response effects of the intervention. 

Similar to other after-school physical activity interventions [12-14], we expect participants would have significantly increased their physical literacy and moderate- to vigorous-intensity physical activity. Few school-based interventions have resulted in increases of fruit and vegetable consumption [20-22]; therefore, this study will add to the literature by evaluating a novel nutrition intervention. Since the access and availability of fruits and vegetables is a predictor of consumption [19], we expect that youth would have significantly increased their consumption of fruits and vegetables at the first and second follow-ups compared to the baseline testing. We also expect greater increases in physical literacy and physical activity with greater attendance at the physical activity programming. In recent years, physical literacy has emerged as a core construct within public health [15]. Therefore, our findings will contribute significantly to the scholarship regarding interventions to increase physical literacy and the association between physical literacy and increased physical activity.

Adopting healthy behaviors in adolescents is important for maintaining healthy behaviors as an adult. However, adolescents in the United States are increasingly inactive and do not meet fruit and vegetable consumption recommendations. The “Move More, Get More” program has the potential to reach large proportions of a student body, unlike competitive sports, which is often the norm. Interventions accessible to all students at a school may be one strategy to increase physical activity and nutrition behaviors for middle schoolers.

### Limitations and Strengths

This study design has several limitations. First, schools were selected based on student need and demographics and were unable to be randomized into the intervention or control group. Consequently, the findings may be due to differences among school or student characteristics rather than the intervention. This limitation highlights the importance of documenting issues related to these characteristics and the need for a process evaluation plan. This study is also limited by relying on several self-report measures and by the measures’ ability to detect variability. For example, nutrition measures asked participants if they consumed the target variable yesterday and provided the following response options: yes, no, and not sure. These questions were required by the grantor, and, to limit the questionnaire size, we did not add additional measures that could potentially detect more variability and changes in participant behavior.

A major strength of this study is that the intervention was developed based on the results of our formative research via focus groups with both middle schoolers and parents, which included the middle schoolers and parents that attend the targeted middle schools [16]. Other strengths of this study include the potential to recruit a large, diverse sample. Further, this study sample is specific to middle schoolers, a population that is under-studied but represents a pivotal time in an adolescent’s development of health behaviors. Lastly, this study used accelerometers to objectively measure physical activity as opposed to relying on student self-report data, which may be biased.

### Future Directions

If the intervention is proven to be effective to increasing physical literacy, physical activity, and fruit and vegetable consumption, our future research will focus on the reach and sustainability of the intervention. Further research is needed on how physical activity interventions are implemented and scaled [39-42]. This study can serve as a model for local and national programming to tackle childhood obesity.
